# Social Media and Youth Mental Health: Scoping Review of Platform and Policy Recommendations

**DOI:** 10.2196/72061

**Published:** 2025-06-20

**Authors:** Jasleen Chhabra, Vita Pilkington, Ruben Benakovic, Michael James Wilson, Louise La Sala, Zac Seidler

**Affiliations:** 1 Centre of Youth Mental Health Orygen Youth Health Footscray Australia; 2 Movember Foundation East Melbourne Australia

**Keywords:** social media, mental health, young people, recommendations, regulation, platform design, policy, artificial intelligence, AI

## Abstract

**Background:**

High rates of social media use and mental ill-health among young people have drawn significant public, policy, and research concern. Rapid technological advancements and changes in platform design have outpaced our understanding of the health effects of social media and hampered timely evidence-based regulatory responses. While a proliferation of recommendations to social media companies and governments has been published, a comprehensive summary of recommendations for protecting young people’s mental health and digital safety does not yet exist.

**Objective:**

This scoping review synthesized published recommendations for social media companies and governments in relation to young people’s (aged 12-25 years) mental health. A qualitative approach was used to undertake inductive content analysis, where recommendations were grouped under conceptually similar themes.

**Methods:**

We searched academic (PubMed, Scopus, and PsycINFO) and nonacademic (Overton and Google) databases for relevant documents. Eligible documents provided recommendations to regulators and social media companies that pertained to social media, young people, and mental health. This review excluded recommendations for young people, caregivers, educators, or clinicians surrounding strategies for managing individual social media use; instead, the recommendations emphasized the regulation or design of social media products and practices of social media companies. Peer-reviewed and gray literature from selected Western contexts (Australia, Canada, the United Kingdom, and the United States) were relevant for inclusion. Documents were published between January 2020 and September 2024.

**Results:**

Of the identified 4980 unique reports, 120 (2.41%) progressed to full-text screening, and 70 (1.41%) met the inclusion criteria. Five interrelated themes were identified: (1) legislating and overseeing accountability, (2) transparency, (3) collaboration, (4) safety by design, and (5) restricting young people’s access to social media.

**Conclusions:**

This review emphasizes the need for multipronged approaches to address the rapidly increasing presence and reach of social media platforms in the lives of young people. These recommendations provide practical and tangible paths forward for governments and industry, backed by expert organizations in youth mental health and technology regulation at a time when expert-informed guidance is sorely needed. Rigorous evaluation of the proposed recommendations is needed while continuing to build on the emerging peer-reviewed evidence base that should form the foundation of policy and regulatory changes.

## Introduction

### Background

The impact of social media on young people’s mental health has emerged as a pressing and global issue of broad public interest, captivating the attention of policy makers, caregivers, educators, and researchers. In Western nations, high rates of social media use among young people indicate the extent to which social media has become a central component in their daily lives, development, and social experiences. In Australia, 98% of young people regularly use at least one social media platform [1]. Similar statistics are observed in other countries, such as the United Kingdom, where an estimated 92% of young people are active on social media by the age of 12 years [2]; the United States, where 95% of those aged 13 to 17 years report using social media [3]; and Canada, where 91% of those aged 15 to 24 years use social media [4].

The definition of “young person” varies across cultural and societal contexts. Following established consensus, we define “young people” as individuals aged 12 to 25 years [5]. This is a time of significant transition, where individuals negotiate and develop their identities, relationships, goals, and values, all of which interact with mental health and well-being [5]. Social media undoubtedly plays a key role in young people’s daily lives as they increasingly use it to shape their identities and forge new connections [6,7]. Young people consistently report social and personal benefits of social media. Social media can be instrumental in maintaining their offline relationships, expanding their social networks, and fostering a sense of belonging [7,8]. Social media also offers opportunities for identity exploration, providing connection among groups that experience marginalization, such as lesbian, gay, bisexual, transgender, and queer (LGBTQ+) communities [9,10], and can facilitate access to information that may not be available or accessed through other means [2,11].

### Young People’s Mental Health and Social Media Content

The social and personal affordances of social media may translate into various mental health benefits. Mental health is a broad, multifaceted construct that encompasses an individual’s emotional, psychological, and social well-being [12]. The social aspects of these web-based platforms, such as the ability to message others and share content, have been linked to greater well-being, reduced depression, increased positive emotions, and decreased loneliness [13-17]. Being connected to support groups can also help young people feel validated and understood and improve their ability to manage their mental ill-health (an umbrella term that encompasses a continuum from the most commonly occurring to the most severe and disabling mental health problems [18]) or suicidal thoughts and behaviors [19]. The often entertaining and motivational nature of certain content can also provide an outlet for young people to relax, have fun, and learn new information or skills, thereby increasing their emotional and psychological well-being [20]. However, such benefits are not universally recognized by others in young people’s lives. In recent Australian surveys of 631 parents and 921 young people (aged 16-25 years), parents ranked social media as the leading concern in relation to their children’s well-being, whereas young people ranked social media as the 24th most pressing concern regarding their well-being [21,22]. This exemplifies a generational disconnect where young people often cite important benefits of social media, whereas caregivers and other adults focus to a far greater extent on its potential harms.

Exposure to harmful content and interactions on social media has increasingly been highlighted as a significant risk factor for young people’s mental health [2,23]. For example, exposure to content related to self-harm and suicidality has been associated with higher rates of depression and anxiety [24] and, in some cases, has been linked to suicide attempts in young people [25]. Exposure to disordered eating content, particularly among adolescent girls [26,27], has been associated with poor self-esteem [28], body image concerns, and eating disorders [29-31]. Cyberbullying, a prevalent form of harassment on social media that is reported by 44% of Australian young people [32], is associated with depression, anxiety, and suicidal ideation [2,24,33].

Given their capacity for social connection, social media platforms can become sites for predatory behaviors and interactions, including distribution of child sexual abuse material and adults seeking to sexually exploit children [34,35]. Exposure to such content can have severe implications for young people’s emotional and psychological well-being, including potentially leading to posttraumatic stress disorder [36] and acute stress disorder [37]. In addition, social media platforms can be used to amplify misinformation (false, misleading, or inaccurate information spread without deliberate intent to deceive) and disinformation (false information deliberately spread to mislead or deceive, which can include manipulated facts, narratives, and propaganda) [38]. Both misinformation and disinformation can jeopardize young people’s understanding of reality, resulting in confusion, fear, and paranoia, and perpetuate harmful attitudes and beliefs [39]. For example, the spread of health-related misinformation (eg, overstating the risks associated with COVID-19 vaccination [38]) has been associated with poor vaccine uptake and mental ill-health in young people (eg, depression, social anxiety, and loneliness [40]). Indeed, young people’s exposure to extremism and hate speech on social media has been linked to the spread of radical ideologies and extremist views [41,42]. Exposure to such content can impact mental health by creating a sense of alienation from peers and communities, resulting in isolation, anxiety, and even violence [43], necessitating platform features for inhibiting this harmful content.

Social media companies use a range of strategies for moderating posts to limit the proliferation of harmful content on their platforms, including human-facilitated and automated methods (eg, artificial intelligence [AI] tools [44]). However, there are concerns that users can easily circumvent moderation practices given that moderation tools can fail to detect harmful posts and have been outpaced by the increasing use of AI and bots on social media [45-47]. These limitations can result in the proliferation of harmful content that may pose direct risks to the mental health of young people.

### Young People’s Mental Health and Social Media Design

In addition to harmful posts on social media platforms, some platform design features may inherently impact young people’s mental health. Rapid advancements in the development and adoption of content recommender systems (a platform design that organizes, prioritizes, and promotes content into users’ feeds based on user actions, preferences, and demographics [48]) have also raised concerns [49]. Recommender systems and highly engaging design features such as “endless scrolling” (a listing page design approach that loads content continuously as the user scrolls down [50]) are used by social media companies to keep users engaged with their platforms for extended periods [51]. Moreover, recent changes to recommender systems have resulted in deprioritizing content from family, friends, and the accounts that users follow in favor of algorithmically curated content [52]. These curated, highly personalized feeds, which are shaped by user data, are designed to keep users on platforms as long as possible [53]. Some suggest that this design feature can amplify young people’s exposure to extreme viewpoints (eg, misogyny, homophobia, racism, or eating disorder content [54]), inappropriate advertising material (eg, promotion of cosmetic procedures and diet supplements), and disinformation [55]. Importantly, engaging app features and curated content have been associated with sleep disturbance, compulsive behaviors (eg, continuously refreshing social media apps), depression, and anxiety, resulting in overall poor mental health and well-being [56,57]. The role of recommender systems in young people’s routine exposure to harmful content is particularly concerning given evidence of rapid exposure to such content despite minimal user engagement. For example, a report [58] found that faux YouTube accounts designed to reflect the demographic characteristics of male users aged 12 to 20 years were frequently exposed to harmful content that promoted restrictive masculine norms and antifeminist rhetoric even in the absence of user engagement, such as watching videos, liking content, or subscribing to channels. This highlights that, even with minimal user input, and in the absence of effective moderation, potentially harmful content can be rapidly pushed to young people’s feeds.

Legislation by governments and regulators such as the eSafety Commissioner in Australia [59], Ofcom in the United Kingdom [60], the Federal Communications Commission and Federal Trade Commission in the United States [61,62], and the Canadian Radio-television and Telecommunications Commission in Canada [63] regulate social media platforms, including their design features, to minimize negative mental health impacts on young people [64]. However, rapid advancements in technology have led to an “arms race” [65] between regulators and social media companies, where the pace of technological advancements and sophistication of algorithms have eclipsed the capacity for timely updates to safety and regulation [64,66]. Moreover, the lack of specificity in existing legislation (eg, allowing legal but likely harmful content such as targeted advertisements [67]) and the lack of international consistency in legislation [64] further complicate efforts to minimize negative mental health impacts on young people. Consequently, there are growing concerns that the largely autonomous governance strategies adopted by social media platforms, coupled with the limitations of current legislations, are insufficient to mitigate the risks to young people’s mental health. As a result, there are calls for stricter regulation, legislative reform, and greater oversight of social media companies’ practices [68,69]. One approach to mitigate negative impacts of social media on young people that is being pursued in some Western countries is restricting social media access. For example, the Australian government has recently legislated restriction of young people’s (aged <16 years) access to social media from 2025 [70]. Similar measures are being discussed in both the United States and the United Kingdom, with the United Kingdom considering the potential restriction of young adults’ access to social media [71].

### This Review

Growing public concern and recent developments in legislation have led to a proliferation in reports and policy documents offering recommendations to governments and social media companies regarding strategies for more effective social media regulation, design, and practices, with an emphasis on safeguarding young people’s mental health. While this is a promising development, the academic literature presently lacks a comprehensive synthesis of recommendations. Therefore, this scoping review aimed to consolidate existing recommendations regarding how social media can be effectively designed and regulated to prioritize the protection of young people’s mental health and minimize harm. This review synthesized available recommendations directed toward governments, regulators, and social media companies using thematic analysis to group them into categories reflecting similar aims or principles.

## Methods

### Search Strategy

This review and reporting methodology followed the PRISMA-ScR (Preferred Reporting Items for Systematic Reviews and Meta-Analyses extension for Scoping Reviews) statement [72]. The search strategy was prepared in consultation with a university librarian. Included documents were identified through 2 sources (Overton and Google) using the following search terms: “Social Media” AND “Young People” AND “Mental Health” AND “Recommendations” OR “Guidelines.” Given the focus on practical, policy-oriented recommendations in this review, the search strategy primarily drew on gray literature sources. Although peer-reviewed literature was eligible for inclusion (and was also considered and trialed when developing the search strategy but yielded few relevant documents), it was deemed more appropriate to prioritize gray literature documents that offered tangible recommendations to governments, regulators, and members of the social media industry, such as policy briefs and reports from nongovernment agencies and advocacy bodies. Overton was chosen as it is the world’s largest repository of policy documents, guidelines, think tank publications, and working papers [73] and, therefore, was particularly relevant to our focus on documents published primarily outside of academic journals. Broad search terms were used to conduct this search as both Overton and Google automatically interpret the terms and use synonyms to capture relevant documents. Additional documents included were extracted from the reference lists of the identified documents or those recommended by expert colleagues. To ensure a comprehensive search of the literature, we reran our search strategy in the following academic databases—PubMed, Scopus, and PsycINFO—but no additional relevant results were identified.

### Selection Criteria

To be included in this study, documents were required to (1) focus on young people between the ages of 12 and 25 years; (2) present recommendations for social media companies, governments, or regulators that promote safe social media products and services for young people; (3) be peer-reviewed published journal articles (original research), case studies, or gray literature (eg, policy briefs and reports); (4) be published in Australia, Canada, the United Kingdom, and the United States or published by global organizations (eg, Amnesty International or the World Health Organization); (5) be published between January 2020 and September 2024 (due to the proliferation of social media platforms and their users after the COVID-19 pandemic [74]); and (6) be published in English.

### Data Screening

Search outputs were imported to the Covidence systematic review software (Veritas Health Innovation) [75]. The first 2 authors independently screened all titles and abstracts against the inclusion criteria. Results were compared, with a disagreement rate of <2% recorded (Gwet AC1=0.97; Cohen κ=0.54). Discrepancies were discussed until consensus was reached between the authors. For the second stage of the review, dual screening was conducted on 25% of the full-text documents. Having achieved 0% disagreement rate, single-author screening proceeded thereafter, which was then checked for accuracy by the second and third author.

### Data Extraction and Synthesis

The extracted data included title, authors, organization, year, country, type of document, target outcomes, and target audience. Information such as recommendations to social media companies (eg, improved platform design) and government bodies (eg, strengthening existing legislations) was extracted. To collate and synthesize the recommendations provided, all documents were exported to NVivo (version 14; QSR International) [76]. The first and second authors analyzed the included recommendations, grouping them into preliminary themes and subthemes to capture conceptually similar ideas. The preliminary themes were then reviewed by the wider coauthor team, and through a collaborative process, the thematic structure was refined to ensure conceptual clarity, coherence, and alignment with the study aims.

## Results

### Overview

**Figure 1:**
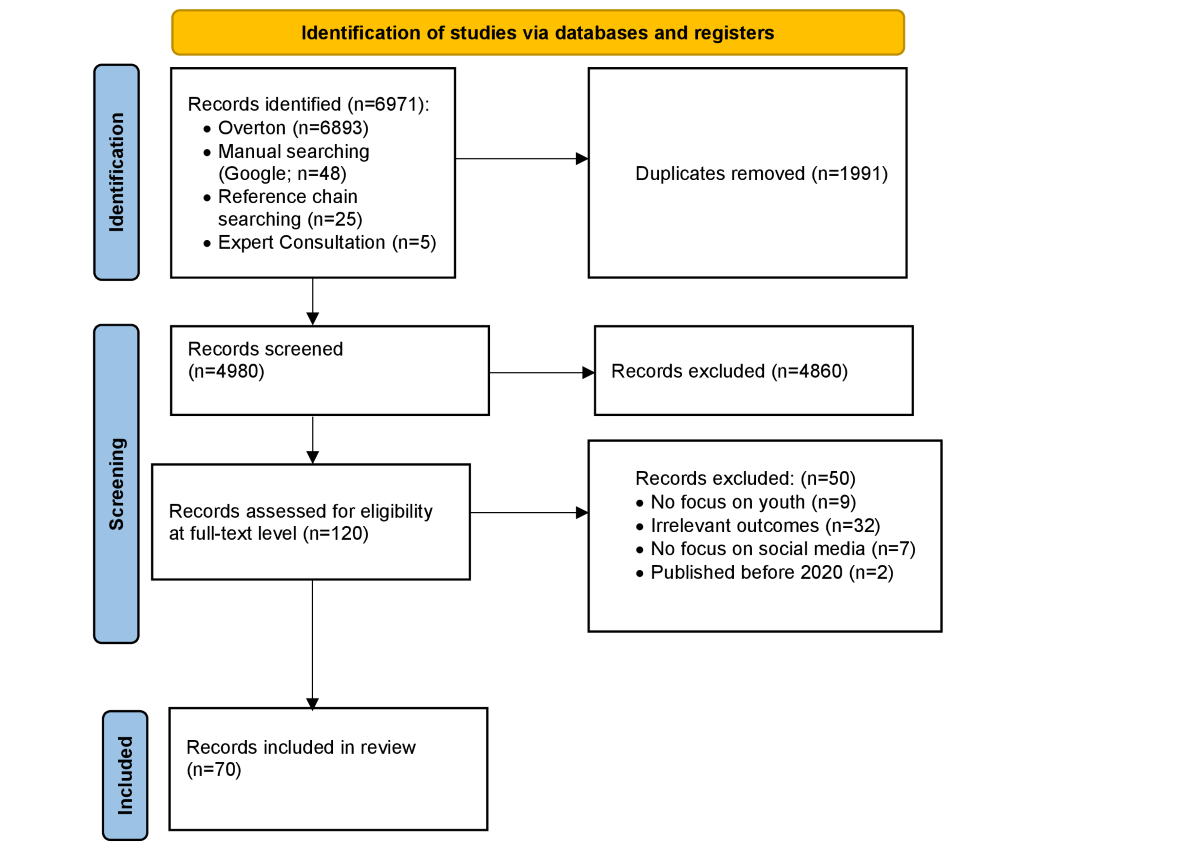
PRISMA (Preferred Reporting Items for Systematic Reviews and Meta-Analyses) diagram.

The search strategy returned 6966 reports, of which 1911 (27.43%) were duplicates. In the initial screening stage, 4980 reports were assessed for relevance. Following the exclusion of 97.59% (4860/4980) of nonrelevant documents, 120 reports progressed to full-text screening, leading to the inclusion of 70 (58%) reports in this scoping review (Figure 1).

### Report Characteristics

Of the 70 included reports, 17 (24%) provided recommendations to regulators or governments, 14 (20%) provided recommendations to social media companies, and 39 (56%) provided recommendations to both regulators or governments and social media companies. The reports sought to target the following outcomes for young people: mental health and well-being related to social media use (35/70, 50%), including exposure to content related to self-harm and suicidality (7/70, 10%); exposure to misinformation and disinformation (16/70, 23%); exposure to discrimination, hate crime, and hate speech (6/70, 9%), including extremist content (9/70, 13%), misogynistic content (5/70, 7%), and cyberbullying (3/70, 4%); exposure to content that increases risk of body image concerns and disordered eating (8/70, 11%); and exposure to pornography and digital child sexual abuse and exploitation (9/70, 13%). Most reports were published in Australia (22/70, 31%), the United Kingdom (18/70, 26%), and the United States (17/70, 24%). The characteristics of the included reports are shown in Multimedia Appendix 1 [3,10,31,33,39,41,52,54,56,58,65,69, 77-134].

### Synthesis of Evidence

**Figure 2:**
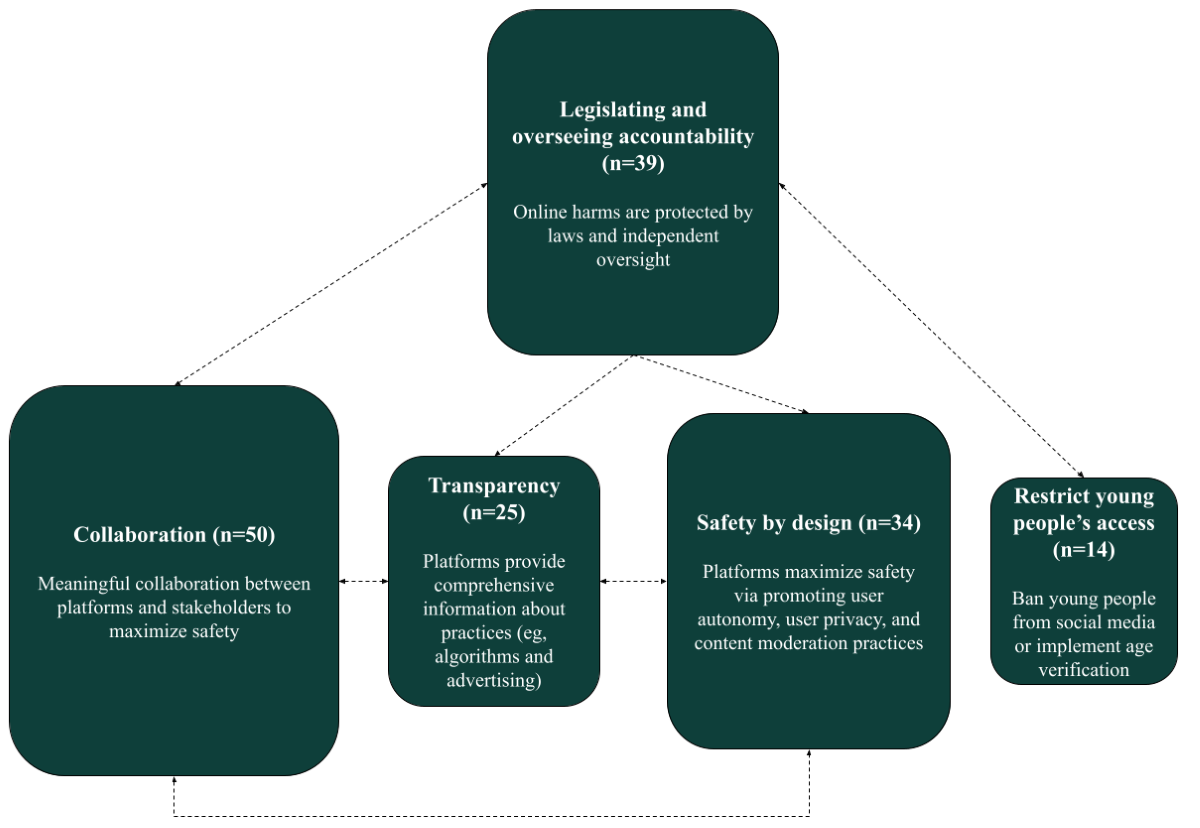
Thematic map of the 5 core interrelated themes of social media recommendations. Note: The size of each box corresponds to the frequency at which each theme was present in the included reports, with larger size indicating that the theme was present in a higher number of reports.

The content analysis resulted in the identification of 5 core interrelated themes, as shown in Figure 2. A PRISMA-ScR checklist was also completed to demonstrate the methodological rigor of this evidence (Multimedia Appendix 2). In total, 4 of the 5 themes centered on strategies for social media companies and regulators to facilitate the provision of safer products to young people, whereas 1 theme centered on restricting young people’s access to social media altogether.

The 5 themes are described briefly in Table 1 and in further detail in the following sections, with illustrative quotes throughout.

**Table 1:** Description of the themes and subthemes.

Theme	Subthemes
Theme 1: legislating and overseeing accountability (n=39)—recommendations regarding the need for governments and independent regulatory bodies to hold greater levels of responsibility and accountability to ensure that (1) users are not harmed; (2) when users are harmed, the harms are addressed appropriately; and (3) failure to address harmful content leads to penalties for social media companies	Creating or reforming legislation—legislative changes to facilitate ensuring that social media companies are accountable in protecting user safety Expanding the definition of online harms—broadening the current definitions of online harms, including incorporating harms associated with body image and technology-facilitated gender-based violence Empowering and equipping regulators—strategies for ensuring that regulators are empowered and equipped to hold social media companies accountable
Theme 2: transparency (n=25)—recommendations on the ways in which social media companies can facilitate access to clear information about their products and practices (eg, with users, governments, and researchers)	Advertising and algorithmic transparency—social media platforms should provide users and others (eg, regulators and researchers) with clear information regarding promotional material on users’ feeds and how users’ algorithms are shaped Transparency reports—social media platforms should release regular prescriptive transparency reports that offer regulators and the public clear and detailed information about content moderation policies and practices, accuracy and error rates for automated review, and number of “take-down” orders, among other things
Theme 3: collaboration (n=50)—recommendations suggesting that social media companies collaborate with relevant stakeholders (eg, parents, caregivers, regulators, and researchers) at all stages of service design and delivery	—^a^
Theme 4: safety by design (n=34)—recommendations on how social media companies can design, develop, and deploy products that are safe to individuals using their services or platforms; includes handling user data and ensuring that potential harms to users are minimized, identified, and addressed	Privacy—recommendations to enhance user privacy and data protection legislations to limit the collection and use of user data by social media companies Content moderation—social media companies’ roles in determining content that is appropriate and safe to appear on their platforms and actions taken to prevent harmful digital content from appearing and proliferating on these platforms and remove it Autonomy—strategies adopted by social media companies (including those responsible for designing, developing, and delivering services) to increase platform users’ decision-making about their (the users’) experiences on social media platforms
Theme 5: restricting young people’s access to social media (n=14)—recommendations regarding implementing age verification measures and banning young people from accessing social media platforms or services until they reach specified ages	—

^a^Not applicable.

### Theme 1: Legislating and Overseeing Accountability

#### Overview

The first theme, legislating and overseeing accountability, encompasses recommendations regarding the need for governments and independent regulatory bodies to hold greater levels of responsibility and capacity to effectively oversee the practices of social media companies. The accountability is to ensure that (1) users are not harmed; (2) when users are harmed, the harms are addressed appropriately; and (3) failure to address harmful content leads to penalties for social media companies. As such, these recommendations aim to enshrine user safety and the accountability of social media companies in law and empower regulators to enforce this with consequences for breaches of liability. Theme 1 contained 3 subthemes, which are discussed in the following sections.

#### Theme 1a: Creating or Reforming Legislation

The subtheme *creating or reforming legislation* appeared in 20% (14/70) of the reports and referred to legislative changes required to ensure that social media companies are held accountable for protecting young users’ mental health. Many recommendations centered on making social media companies liable for allowing dissemination of harmful user-generated content on their platforms [77]. This included imposing penalties “sufficient to realise the goal of deterrence” [78]. Some reports (8/14, 57%) referred broadly to “harmful material” posted on social media platforms, whereas others (6/14, 43%) specifically discussed the need to impose liabilities regarding content that encourages extreme dieting, eating disorders, self-harm [79], and violent crimes [78] (quote from Canadian Commission on Democratic Expression [77]):

Legislation can and should leave open the possibility of platforms being held liable for violating their duty to act responsibly in relation to the algorithmic curation and amplification of certain kinds of content.

A number of reports (2/14, 14%) recommended changes to Section 230(c)(1-2) of the 1996 Communications Decency Act in the United States [78,80], which currently offers social media companies immunity from responsibility for user-generated content on their platforms. With the amendment of this law, regulators would be able to hold owners of platforms such as TikTok and Instagram (eg, Meta and ByteDance) accountable for harmful posts (eg, related to self-harm or hate speech) posted and shared by users. Implementing such legislative changes, with associated penalties for noncompliance, would likely encourage platforms to enact more effective self-regulation [80] (quote from Office of the New York State Attorney General Letitia James [78]):

Instead of simply being able to assert protection under Section 230, a defendant company has the initial burden of establishing that its policies and practices were reasonably designed to address unlawful content.... This would help establish a baseline of content moderation across online platforms, helping to ensure a safer experience for all users.

#### Theme 1b: Expanding Definitions of Online Harms in Legislation

The subtheme *expanding definitions of online harms* referred to recommendations in 13% (9/70) of the reports regarding broadening current definitions of online harms in relevant legislation (eg, Australia’s Online Safety Act). Amending this definition was suggested to ensure consistent and comprehensive prevention and response measures regarding content on social media platforms deemed likely to cause harm to users [81].

Several specific online harms were noted in the included reports. For example, the Women and Equalities Committee [82] called for inclusion of appearance-based bullying in UK legislation pertaining to online harms; the Butterfly Foundation and Daniel [81] recommended inclusion of harms associated with body image in the Australian Online Safety Act; and the Royal Society for the Encouragement of Arts, Manufactures, and Commerce [83] suggested that the Online Safety Act include harms caused by misinformation in its remit. Furthermore, one report that focused on extremism and hate speech on TikTok suggested that the platform should offer a clear definition of extremism in their terms of service, arguing that effective restriction of harmful material hinges on clear definitions of harm [41] (quote from The Butterfly Foundation and Daniel [81]):

The Online Safety Act 2021 (Cth) should be modified so that members of the Australian public can report to the eSafety Commissioner when they see material that could negatively affect their body image.

The Institute for Strategic Dialogue (ISD) [39] reflected on the need for legislation to adapt to emerging virtual reality spaces, including what constitutes crimes within the Metaverse. The ISD [39] argued that uncertainty about how offenses such as sexual assaults occur within these emerging digital spaces warrants the need to review existing criminal codes, define criminal behavior in virtual spaces, and ensure that legislation exists to prosecute such offenses (quote from Dorn et al [39]):

It should be argued that the immersiveness of the Metaverse can result in the very real (emotional) experience of rape, reiterating questions about the requirements of existing laws to quality for the legal terms of rape.

#### Theme 1c: Empowering and Equipping Regulators

The subtheme *empowering and equipping regulators* appeared in 43% (30/70) of the reports and referred to the need to implement strategies that ensure that independent regulators are equipped to hold social media companies accountable for ensuring young users’ safety and addressing any harms encountered when young people use social media.

Specific recommendations included conducting routine independent audits [84], establishing independent regulators (including data protection regulators [85]), and ensuring that regulators have power to impose penalties when social media companies fail to comply with relevant rules and regulations [65,77]. Within these reports, the authors highlighted the importance of external oversight in light of the current norm of self-regulation of practices and policies by social media companies [84-86] (quote from Amnesty International [85]):

Stronger laws and regulation on data protection and algorithmic amplification of content on social media, and effective enforcement of such laws and regulation, are needed in order to keep children safe from the harvesting and exploitation of their personal data for profit.

Several reports (7/30, 23%) recommended establishing new independent regulators focused on auditing, investigating, and suggesting reforms to the policies and practices of social media platforms and their enforcement [77,78]. The authors noted the need for such regulators to have information-gathering powers and access data held by social media companies for oversight purposes [65,77]. This also requires regulators to be appropriately informed and equipped to deal with the complex, ever-changing nature of digital technologies and behavior [87] (quote from Reset Australia [65]):

To meaningfully drive change, regulations need to be enforceable, and regulators must be empowered and resourced.

### Theme 2: Transparency

#### Overview

The second theme, *transparency*, encompassed recommendations regarding ways in which social media companies can increase the clarity of information regarding their products and practices. This includes communicating with users, governments and regulators, and researchers. This theme contained two subthemes: (1) *advertising and algorithmic transparency*, which related to transparency about what users see on their platforms or feeds; and (2) *transparency reports*, which related to the need for consistent and prescriptive official reporting of social media companies’ practices and policies, particularly regarding content moderation.

#### Theme 2a: Advertising and Algorithmic Transparency

Calls for advertising and algorithmic transparency appeared in 23% (16/70) of the reports. These recommendations emphasized the importance of offering greater transparency about what users are exposed to when using social media platforms. These recommendations argue that social media companies should provide users with clearer information regarding promotional material on users’ feeds and how algorithms are shaped.

Development of advertising repositories, or openly searchable databases of all advertisements presented on social media platforms, was suggested in several reports (5/16, 31%) [65,69,87]. It was recommended that these repositories include clear details about the advertiser, advertisement content, targeting parameters (ie, specific demographics of the users being targeted), demographics of those exposed to advertisements, and the advertising spending and length of campaigns [77,84,87]. Several reports (3/16, 19%) noted a need for greater transparency surrounding political advertising in particular [77,84,87]. For example, during the US 2024 elections, several reports (3/16, 19%) suggested that disinformation on platforms such as X (formerly known as Twitter) and Facebook heavily influenced individuals’ views of candidates and their policies on the economy, immigration, and crime [135] (quote from Canadian Commission on Democratic Expression [77]):

Advertising transparency will aid in the identification of discriminatory and biased advertising practices...Even advertisers would benefit from transparency that provides them with more data about how platforms manage their advertisements, details about views and clicks on the advertisement and the influence of bots rather than humans, than is currently made available to them.

Recommendations also advocated for transparency regarding algorithms, where social media companies should articulate to users how their algorithms (or “feeds”) are shaped [81,88]. The authors noted that social media algorithms operate “opaquely, with little transparency regarding how content is prioritised and served to users” [52] (quote from the Jed Foundation [89]):

[We recommend] Supporting federal regulation designed to maximise protective factors, including...promoting transparency in algorithms across social media platforms, ensuring that young social media users gain insight into the factors shaping their online experiences.

Recommendations also pertained to social media influencers and celebrities. These included calls for these figures to be made subject to stricter regulation, ensuring that any promotional, paid, or incentivized posts are clearly signposted to users [31]. It is important to acknowledge that, currently, promotional or paid posts are often labeled as “paid.” However, the rapid increase in the number of social media influencers, their followers (young people), and the promotional materials presents new challenges for advertising regulators, requiring stricter disclosure and transparency standards [82].

#### Theme 2b: Transparency Reports

A total of 23% (16/70) of the reports called for transparency reports to be released regularly by social media companies, offering clear and publicly accessible information about social media companies’ policies and practices, particularly surrounding content moderation and removal. It was acknowledged that transparency reports have begun to be released by key platforms, although the authors noted the need for clear rules regarding what should be included in these reports to facilitate *meaningful* transparency [84-86,90]. In particular, the Office of the New York State Attorney General Letitia James [78] and Reset Australia [84] offered a number of tangible recommendations regarding what should be included in social media companies’ routine transparency reports (quote from Reset Australia [84]):

Annual transparency reports are necessary, that include clear prescriptions around information that must be shared to create meaningful transparency. It also helps create the conditions to compare services and track changes over time in meaningful ways.

Recommended components included details about (1) the *detection* of content deemed to violate social media companies’ community guidelines (eg, number of posts within a specified period, number and proportion detected through automated means, and number and proportion reported by users); (2) the accuracy and error rates for automated review processes; (3) the *types* of content reported and moderated on social media platforms, including specific types of violations of community guidelines (eg, self-harm and child sexual abuse material); (4) the *responses* of social media companies when content is deemed to violate their community guidelines and relevant regulations (eg, time taken for posts to be removed); (5) the number of *take-down orders* issued by regulators and social media companies’ responses to these orders (eg, action taken by the company and response times); and (6) the number of out-of-court settlements made.

### Theme 3: Collaboration

The third theme, *collaboration*, was documented in 71% (50/70) of the reports and included recommendations that social media companies collaborate with important stakeholders at all stages of service design and delivery. This included social media companies consulting and collaborating with governments, relevant agencies, young people, health practitioners, and researchers.

In some reports (12/50, 24%), the authors advocated for investment in research regarding possible impacts of social media on young people’s mental health, well-being, and brain development [52,91]. The need for research funding was highlighted in 24% (12/50) of the reports [92-94], along with acknowledgment that conducting such research requires cross-sector cooperation and facilitating researcher access to social media user data. Indeed, several reports (37/50, 74%) highlighted that enhanced collaboration (ie, among social media companies, researchers, and policy makers) will require wider data sharing agreements [33,65,77] (quote from ReachOut, Beyond Blue, and Black Dog Institute [52]):

Ensuring that young people’s experiences, agency and voices are central to policy development will lead to more relevant and effective interventions.

Several recommendations emphasized the need to better understand the digital experiences and needs of specific vulnerable groups, who are often at risk of digital harms [86]; hear from a diverse range of voices; and prioritize cross-cultural collaboration [95,96]. For example, this includes engaging with young boys who encounter misogynistic content in digital spaces [95] in addition to engaging with young people from ethnic minority groups, LGBTQ+ young people, and young people experiencing mental ill-health [97]. As outlined in the following quote, marginalized young people may be particularly vulnerable to digital harms; however, they may also find highly affirming networks on these digital spaces that *support* their well-being (quote from Madden et al [97]):

Youth, especially youth of color, LGBTQ+ youth, and young people with depressive symptoms, are in need of safe spaces, support, and resources both online and offline. Young people who turn to social media for support and search for resources online suggest that their offline communities and environments do not provide enough for their needs...

Some reports (7/50, 14%) highlighted the importance of communication and consistency *between* different social media platforms given that the vast majority of users use multiple platforms [41,78]. This includes cross-sector research and collaboration (eg, developing protocols for age restriction; see theme 5), which should be subject to independent evaluation and auditing (quote from O’Connor [41]):

Partnerships between companies can help to recognise and react to known terrorist threats more quickly than otherwise possible and reflect the cross-platform nature of online threats. However, any such effort should be transparent and clearly defined, making the objectives, processes and outcomes of such collaboration clear from the outset, with opportunities for auditing and evaluation by independent experts from civil society and the research community.

### Theme 4: Safety by Design

#### Overview

The theme safety by design outlines recommendations on how social media companies can design, develop, and deploy products that are safe for young users of their services and platforms. This includes the collection and use of user data and ensuring that potential harms to users are minimized, identified, and addressed. This theme contained 3 subthemes: *privacy*, *content moderation*, and *autonomy*, each of which represents key areas of responsibility for social media companies to deliver safe products to end users.

#### Theme 4a: Privacy

In total, 21% (15/70) of the reports highlighted the importance of social media companies having suitable processes, policies, and actions regarding the collection and use of user data. Several reports (11/15, 73%) strongly recommended enhanced and updated privacy (eg, establishing the user privacy settings to the maximum by default) and data protection legislation to limit the collection and use of young people’s data. This included limiting social media companies in using user data to shape personalized algorithms and advertising to young people [33,77,98,99]. Such legislation could support data minimization (collecting the minimum amount of personal user data to deliver services), reduce personalized content, and create special protections for particularly sensitive data (eg, biometric information and precise location data), particularly for young users [98,99].

#### Theme 4b: Content Moderation

A total of 26% (18/70) of the reports explored recommendations for content moderation policies, practices, and design features used by social media companies. Recommendations advocated for stronger investment in content moderation staffing and technologies [78], increased use of human moderation [33,100], and use of advanced AI for detecting and removing content that violates community guidelines [86]. The moderation of content posted across an array of languages was also recommended [84,86] (quote from Amnesty International [33]):

Ensure consistency in content moderation decision making, ensure adequate human oversight of automated content moderation and appropriate investment in content moderation resourcing across all languages.

It was also recommended that social media companies do more to understand how users evade detection in the current systems (eg, using visual cues such as emojis, replacing blacklisted terms with similar-sounding words, or altering posts from their original forms [41,101]) (quote from ISD [41]):

TikTok must regularly update its list of blocked terms, incorporating a significantly increased number of variations and misspellings, and apply these filters not just to search results but also to hashtag suggestions.

#### Theme 4c: Autonomy

A number of reports (20/70, 29%) provided recommendations pertaining to social media companies increasing the capacity for platform users to make informed decisions regarding their experiences on the platform. Recommendations advocated for safety design features (eg, opting out [102]), allowing young people to be active agents in personalizing their feed or algorithm, including their increased capacity to “suppress” types of content that they do not wish to see on their feeds [33,56,57,89,103]. This design feature may allow users to restrict or limit posts that negatively impact their mental health (eg, disordered eating, misogynistic content, and self-harm) on their feeds [85,94]. Offering young people greater choice about the type of content shown on their feeds is considered a potential protective factor against mental ill-health [89,100] (quote from The US Surgeon General’s Advisory [94]):

Give users [young people] opportunities to control their online activity by opting out of the content they may find harmful...such as ads involving violence, alcohol, or gambling, or content related to eating disorders.

Recommendations regarding specific design features on social media platforms were also offered, for example, allowing users greater choice and control over design features (eg, turning off or disabling the “infinite scroll” feature on TikTok [52,98,100]) and greater choice on how their collected data are used (eg, providing informed consent [77,85]). This enhanced user choice may allow young people to use social media platforms in ways that better meet their needs and preferences [98]. Others (10/20, 50%) offered stronger recommendations. For example, the Office of the Minnesota Attorney General Keith Ellison [102] proposed that “dark patterns” within platform design that encourage excessive use for young people “beyond their explicit desires”—such as infinite scrolling, auto-play videos, engagement-driven algorithms, and frequent notifications—be banned altogether.

### Theme 5: Restricting Young People’s Access to Social Media

Several reports (14/70, 20%) discussed the potential benefits and drawbacks of implementing age restrictions on young people so that they cannot access social media platforms until they reach specified ages. Some authors (5/14, 36%) recommended the introduction of mandatory age verification for all social media accounts to ensure that users met the minimum age criteria [82,104,105]. However, others (5/14, 36%) highlighted the potential for age verification technologies to exacerbate existing concerns, such as social media companies’ collection and use of large amounts of user data [33,98,99]. For example, introducing age verification and limiting young people’s access to social media may pose further risks to young people [98]. In particular, age verification will likely involve social media companies assuming responsibility for collection of additional personal user information by requiring government-approved IDs (or other similar documentation) to verify user age, therefore posing privacy concerns (quote from Forland et al [98]):

While more eﬀorts are needed to ensure children can safely and securely access online spaces, age veriﬁcation mandates may actually pose more risks than beneﬁts—resulting in unintended consequences for the constitutional rights, privacy, and security of all users.

Thus, recommendations emphasized the necessity for multipronged solutions given that “age verification is no substitute for privacy protections and increased user transparency and control” [98]. Reports also called for evaluation of the effectiveness of age verification technologies as these necessarily mediate any widespread age-based ban [98,100]. In addition, recommendations for alternative approaches to age verification via government IDs were suggested to circumvent privacy concerns [106]. For example, the Australian government suggested taking advantage of an existing service, the Australian Digital ID program, to facilitate age verification in ways that minimize privacy concerns [107]. Other recommendations included involving caregivers in verifying children’s ages [104,105], using facial recognition technologies (eg, Yoti [106]), and collaboration among a range of stakeholders to develop best practices and effective age verification systems to minimize the impact on young users [94,98,107] (quote from Forland et al [98]):

Insights from industry, civil society, regulators, and users of all ages should be taken into consideration to create standardized best practices and protocols for age veriﬁcation.

Finally, recommendations were made to explore alternatives to age verification processes (eg, stronger legislation, empowered regulators, and safety- and security-by-design features [98,104,106,107]) to mitigate the additional privacy and safety concerns (quote from Johnson [106]):

An alternative to age verification would require device operating systems to create a “trustworthy child flag” for user accounts that signals to apps and websites that a user is underage and require apps and websites that serve age-restricted content to check for this signal from their users and block underage users from this content.

## Discussion

### Principal Findings

#### Overview

To our knowledge, this is the first review to synthesize recommendations regarding social media and young people’s mental health. This synthesis is especially timely in the context of ongoing discourse surrounding harms associated with social media, legislative changes regarding young people’s access to social media [70,136], and growing tension between governments and social media companies [137]. This review included 70 reports from Australia, Canada, the United Kingdom, and the United States. The identified themes highlight wide-ranging opportunities for governments, regulators, and stakeholder collaboration in ensuring that social media products are designed and delivered to young people in ways that safeguard their mental health. Moreover, these themes are consistent with published empirical works and reviews [7,8,23,36].

#### Strategies for Limiting Harmful Content on Social Media Platforms

While social media companies have introduced various content moderation tools to reduce harmful content on their platforms [138], many reports (10/18, 56%) highlighted that existing strategies are insufficient to prevent young people from being exposed to digital harms and advocated strongly for improved content moderation practices and policies [30,33,104]. This aligns with empirical evidence that indicates that more than 3 in 4 young people report exposure to digital hate content [4,23] and findings indicating that young people are approximately 4 times more likely to see posts related to self-harm or suicide relative to those aged >25 years [11]. Perspectives on moderation strategies were mixed, with some (6/18, 33%) advocating for increased investment in human moderators [33,100], whereas others (5/18, 28%) recommended greater use of AI-facilitated moderation. This comes in the wake of many social media companies’ retrenchment of content moderation workforces in recent years (eg, Meta’s decision to replace paid moderators with “voluntary community notes” on its platforms [139-142]). However, as noted throughout the literature, overreliance on AI-facilitated moderation and natural language processing techniques warrants careful consideration [44]. AI moderation tools often fail to recognize and remove iterations of banned material (eg, terms such as “unaliving” or hashtags such as #suwerslide instead of #suicide) [88]. Indeed, users routinely circumventing banned words and topics only serves to highlight important gaps in the efficacy of current moderation practices [46]. Notably, the included reports rarely alluded to young people’s *intentional* exposure to potentially harmful digital material, such as posts related to self-harm or suicide. This is a considerable oversight as previous studies have shown that some young people actively seek information and support regarding these digital challenges, particularly given that social media can offer anonymity, cost-effectiveness, and a sense of connection with others experiencing similar hardships [11]. Nonetheless, the need for effective and sophisticated moderation tools to reduce exposure to potentially harmful posts was emphasized across the reports, as has been advocated for widely in the extant academic literature [44,46]. Such action is likely to garner support and foster trust in social media services among the public based on data indicating that the general community is supportive of governments and social media companies taking proactive steps to remove harmful digital content [23]. However, there is also a need to consider strategies for making posts related to mental health available and safe for young people to access on the web [11].

Recommendations also emphasized that social media companies must be held responsible for young users’ exposure to harmful digital content through legislative enforcement of compliance. Strong support was expressed for legislative changes to support ensuring the liability of social media companies for harmful content being created and disseminated on their platforms rather than allowing for immunity [77,78]. Alongside amending and introducing necessary legislation and expanding the definition of online harms [80,108,109], the authors recommended empowering independent regulators with meaningful auditing, investigative, and enforcement powers [65,77].

Crucially, regulations and moderation practices must be fit for purpose and informed by a thorough understanding of digital technologies (including routinely used strategies for nondetection). They should be continually reviewed and adapted to ensure that they are responsive to technological advancements and emerging digital safety issues, such as the use of “bot accounts,” AI-generated content, and encoded file formats, and responsive to changing digital landscapes to avoid the “legal lag” [65].

#### Strategies for Improving Social Media Platform Design

This review captured several recommendations to improve design features that keep young people engaged on social media platforms for long periods, such as infinite scrolling, auto-play videos upon opening platforms, and constant push notifications [56,100]. Given young people’s developmental stage, these features are considered particularly harmful to them [143] and have been associated with feelings of depression, anxiety, hopelessness, and isolation [144,145]. As a result, the reports recommended that social media platforms use human-centered design approaches, where users (including young people) and other stakeholders (eg, caregivers) are involved in platform design [100].

Implementing age verification in social media platforms has been a topic of considerable debate, with Australia recently mandating age restrictions for platforms such as Instagram, TikTok, and Snapchat [10,70]. While some (5/14, 36%) argued that limiting young people’s access to social media is crucial for preventing their exposure to inappropriate content [3,146], others (5/14, 36%) expressed concerns about feasibility and the possible risks that age verification may present [98]. Criticism of age verification included uncertainty about how to effectively implement verification measures, risk of underage users bypassing restrictions, and privacy concerns. For example, if age verification measures require users to provide identification documents to sign up to social media platforms, they may risk further infringements on users’ personal information or serious privacy breaches [147]. Consequently, decisions regarding age restriction and verification require careful consideration regarding how best to ensure user safety, accessibility, and privacy [100].

Age restriction represents one of many possible responses to the perceived risks to young people’s mental health that social media can pose. However, this approach ignores the possible benefits and adaptive uses of social media among young people [7-9], including social connection and self-expression [6]. Indeed, based on existing literature, age restrictions could have significant *negative* impacts on some young people, particularly marginalized groups such as LGBTQ+ and migrant youth [146]. Many of these young people rely on social media to connect with their communities, obtain information they would not seek (or be able to seek) in offline contexts, combat stigma and isolation, and find support [9,11]. Some have also noted that removing young people’s access to social media risks them turning to digital spaces that have less stringent safety measures and pose *greater* risk to mental ill-health, such as unmoderated forums [148]. As such, restrictive approaches have been described by experts and academics as a “blunt tool” [147,149] that does little to ensure that social media products are made safer for all users, including young people. In sum, while some view age restriction as a necessary step toward safeguarding young people, others advocate against this and propose more nuanced approaches—such as implementing and legislating age-appropriate safety-by-design features and content moderation practices. The latter approach may offer a greater balance between safeguarding youth and preserving their access to supportive digital spaces [110,147].

#### Strategies for Increasing Transparency and Collaboration

The need for greater transparency from social media companies was frequently highlighted in the recommendations. This included communicating openly and clearly with governments, regulators, users, and the public about their policies and practices regarding the type of data that are collected from users and how they are used, how algorithms are shaped, what posts constitute paid or promotional content, and the types and frequencies of posts being removed from these digital spaces [110].

Although several studies (10/16, 62%) suggested that social media companies release regular transparency reports [41,87,90], this recommendation has since been implemented in countries such as Australia, the United States, the United Kingdom, and Canada. To combat regulatory scrutiny, social media platforms release quarterly transparency reports that highlight the measures taken to address misinformation and disinformation [84]. However, regulators have raised concerns over the quality and utility of current transparency reports [86]. For example, when figures about violative content are presented (eg, 10,000 posts were removed), these figures are often a combination of many metrics and lack clarity about the *type* of violation for which the posts were removed (eg, posts inciting violence or posts proven to be false after fact-checking [84]). They also do not indicate how many reported posts are not removed, the maximum time until removal, and the scope of the posts’ spread before their removal. Additional information is required to increase the utility of transparency reports, such as the number of take-down orders issued by regulators, median and maximum times until posts deemed to violate community guidelines are removed, and specific actions taken following removal [84,150].

Transparency reports have also been criticized for their lack of objectivity as they are often developed by the social media companies that have a vested interest in the findings. In addition, the social media companies’ refusal to grant data access to researchers prevents independent verification of the claims presented in these reports. Indeed, authors of the included reports argued that providing approved researchers with access to the data collected by social media platforms will enable the evaluation and auditing of content moderation systems, platform policies, and their alignment with policies and regulations [84,87,98]. This echoes similar calls by academics [151]. Researcher access may also enable the auditing of actions taken by social media companies against harmful content and users who violate platform policies [92]. To minimize privacy risks and maximize user safety, access to public data should only be made available to “vetted” researchers [84,151] and will require data sharing agreements in addition to coordination and collaboration between social media companies and relevant regulators [100].

Currently, there is a significant gap in longitudinal research on the effects of social media use on young people, leading to an incomplete understanding of both potential harms and benefits [3]. Indeed, some studies suggest that social media use is *positively* associated with important correlates of well-being such as loneliness [13]. As highlighted in previous reviews, long-term studies are essential to comprehensively assess how particular social media content and behaviors impact youth mental health, development, and behavior over time [7,9]. Academics also increasingly emphasize the importance of considering nuances in *how* young people use social media (eg, which platforms and the types of content viewed) and for what purposes (eg, entertainment, information gathering, and social connection) rather than framing proposed links between social media use and mental health as a linear relationship, wherein increased time on social media necessarily predicts poorer outcomes [6,13]. Furthermore, there is a need to consider whether particular groups are at elevated risk of being impacted by potential digital harms (eg, mental ill-health, exploitation, and disinformation) as this may guide targeted prevention and intervention efforts. Providing researchers with access to real-time data, alongside substantial investment in research, would greatly enhance the ability to conduct such studies, enabling the monitoring of changes and trends in young people’s social media behavior and broader effects over extended periods [92]. Importantly, researchers also recommend that empirical findings be coupled with young people’s insights and perspectives, which can be obtained via qualitative studies and meaningful consultation [6,11]. These approaches would offer more informed conclusions and evidence-based recommendations for managing the impacts of social media on young people [87].

While collaboration among regulators, social media companies, and researchers is a crucial step toward creating safer platforms, young people should remain at the center of this collaboration. Several reports (10/50, 20%) recommended consulting and collaborating with young people, including those who are typically underrepresented during the development of legislation, regulation, and social media platform design [33,39,111]. Existing research shows that policies and programs that involve young people in their development are more likely to be effective and well received [52]. This participatory approach also ensures that the solutions are more relevant and tailored to the specific needs and experiences of young people [52]. Young users are often the first to encounter new and evolving risks on social media platforms. Therefore, their input is invaluable in identifying and addressing these challenges. By incorporating their perspectives, policies can be better equipped to tackle the complexities of digital behavior, mental health concerns, and digital safety, offering more nuanced and practical solutions that align with young people’s lived experiences [52,111].

### Limitations

This review’s findings should be viewed in light of several limitations, including the paucity of non–English-language documents. This limits the generalizability of the results to non–English-speaking contexts and may not account for the impact of social media practice and policy on youth mental health worldwide. While this review focused on countries that have been leading in terms of regulatory efforts and public discourse regarding social media and youth mental health, it is suggested that future international reviews broaden their scope and capture recommendations for governments and regulators and the social media industry at a global level. A global review will also bring forward the issues faced by middle- to low-income countries where young people are more likely to be vulnerable to the negative impacts of social media (eg, lack of resources to circumvent targeted advertising and marketing). In addition, although this is the first review to collate recommendations regarding social media and youth mental health, the recommendations were not categorized based on the developmental stages of users aged 12 to 25 years. This omission was due to the absence of age-specific recommendations in the existing literature. Future research should be mindful of sensitizing recommendations based on user age as blanket policies that attempt to address the unique needs of both those aged 12 years and those aged 25 years under the same regulatory framework may prove ineffective.

This review predominantly captured gray literature, which precluded evaluation of the quality of the reports and contributed to the widespread lack of evidence-based recommendations. Nonetheless, the recommendations were rationally derived by subject matter experts and organizations. Our focus on the available (largely gray) literature represents a pragmatic attempt to synthesize recommendations that are likely to be viewed by governments and regulators in the absence of corresponding and contemporaneous academic literature. Furthermore, given our focus on recommendations for the social media industry and regulators, it was beyond the scope of this review to collate recommendations specifically for young people, clinicians, caregivers, or educators. While we acknowledge that this may have meant that we overlooked some important recommendations for protecting young people’s mental health and digital safety, our focus was intentional to highlight the many and varied opportunities for the social media industry itself, and associated regulators, to enforce safety. While the authors acknowledge that this overlooks some relevant recommendations regarding the relationships between young people’s mental health and their social media use, the goal of this review was to move away from user responsibility and demonstrate the varied opportunities for the social media industry and regulators.

Finally, the recommendations in this review were rarely informed by rigorous peer-reviewed evidence, which requires amelioration as the evidence base proliferates. This limits current understanding regarding the efficacy and impacts of enacting the types of recommendations proposed in the included reports. Rigorous, peer-reviewed studies are needed to systematically evaluate the efficacy and real-world impacts of the proposed recommendations. Strengthening this evidence base will ultimately guide more informed decision-making and policy development.

### Conclusions

This scoping review aimed to provide a synthesis of published recommendations for governments, regulators, and the social media industry at a time when both government bodies and social media companies are considering implementing landmark changes to protect the mental health of young people. The scope of evidence across the 5 interrelated themes highlighted in this review emphasizes the range of options available to both industry and government and the imperative to undertake a multipronged approach. These 5 themes, while distinct, are clearly interrelated and collectively offer a comprehensive array of strategies to address the increasing presence of social media in young people’s lives and likely their health. At a time when the empirical evidence for the harms and benefits of social media in young people is still emerging, the expert-based recommendations in this review establish a tangible path forward for both governments and social media companies to safeguard the health of young people. This review underscores the pressing need for coordinated, evidence-informed action from policy makers and platforms, particularly as discourse surrounding social media and youth mental health continues to intensify and outpace regulatory and industry responses. In this context, the emergence of reactive and restrictive action, such as proposals to ban young people from social media, risks diverting attention from the more complex but essential task of creating safer and more supportive digital environments.

## Appendix

Multimedia Appendix 1 Characteristics of the included studies.

Multimedia Appendix 2 PRISMA-ScR (Preferred Reporting Items for Systematic Reviews and Meta-Analyses extension for Scoping Review) checklist.

## Abbreviations

AI: artificial intelligence
ISD: Institute for Strategic Dialogue
LGBTQ+: lesbian, gay, bisexual, transgender, and queer
PRISMA-ScR: Preferred Reporting Items for Systematic Reviews and Meta-Analyses extension for Scoping Reviews
