# Supplementary material for: Social Media and Youth Mental Health: Scoping Review of Platform and Policy Recommendations
Source: J Med Internet Res. 2025 Jun 20;27:e72061. doi: 10.2196/72061 (PMC12228008; doi:10.2196/72061)
Supplement: Multimedia Appendix 1 [file jmir_v27i1e72061_app1.docx]

**Table S1**. Characteristics of included studies

| **Reference #** | **Organisation** | **Individual authors (if applicable)** | **Year** | **Title** | **Country** | **Target outcome(s)** |
| --- | --- | --- | --- | --- | --- | --- |
| [95] | Advancing Learning and Innovation Gender Norms (ALiGN) | Stephanie Diepeveen | 2024 | How Does Social Media Influence Gender Norms Among Adolescent Boys? | UK | Misogyny |
| [92] | American Psychological Association (APA) | NA | 2023 | Health Advisory on Social Media Use in Adolescence | USA | Mental Health & Wellbeing |
|  |  |  |  |  |  |  |
| [85] | Amnesty International | NA | 2023 | "I Feel Exposed": Caught in TikTok's Surveillance Web | Global | Mental health & Wellbeing, Suicide, and Self-harm |
| [33] | Amnesty International | NA | 2023 | Driven Into the Darkness: How TikTok's 'For You' Feed Encourages Self-Harm and Suicidal Ideation | Global | Suicidal Ideation and Self-harm |
| [56] | Black Dog Institute | Kate Maston, Lyndsay Brown, & Aliza Werner-Seidler | 2024 | Adolescent screen use and mental health: Summary of findings from the Future Proofing Study | AUS | Mental Health & Wellbeing |
| [112] | Boston University | Sandro Galea & Gillian J Buckley | 2024 | Social media and adolescent mental health: A consensus report of the National Academies of Sciences, Engineering, and Medicine | USA | Mental Health & Wellbeing |
| [81] | Butterfly Foundation | NA | 2024 | Social Media, Body Image and Eating Disorders | AUS | Eating Disorders |
| [113] | Center for Countering Digital Hate | NA | 2022 | The Incelosphere | UK | Mental Health & Wellbeing, Self-harm & Suicide, Misogyny, Extremism, Misinformation & Disinformation, Hate Speech |
| [87] | Centre for Data Ethics and Innovation | NA | 2020 | Online targeting: Final report and recommendations | UK | Mental Health & Wellbeing |
| [114] | Centre for International Governance Innovation (CIGI) | Suzie Dunn, Tracy Vaillancourt & Heather Brittain | 2023 | Supporting Safer Digital Spaces | CANADA | Technology-facilitated gender-based violence (TFGBV) |
| [91] | CIGI | Samantha Bradshaw & Tracy Vaillancourt | 2024 | Freedom of Thought, Social Media and the Teen Brain | CANADA | Mental Health & Wellbeing |
|  |  |  |  |  |  |  |
| [80] | Centre on Regulation and Markets at Brookings | Sanjay Patnaik & Robert E. Litan | 2023 | TikTok Shows why Social Media Companies Need More Regulation | USA | Misinformation, Disinformation |
| [115] | Data & Society | Amanda Lenhart & Kellie Owens | 2021 | The Unseen Teen - The challenges of building healthy tech for young people | USA | Mental health & Wellbeing |
| [116] | Department for Digital, Culture, Media and Sport (DCMS) | Perspective Economics, Mary Aiken, & Julia Davidson | 2020 | Safer Technology, Safer Users: The UK as a World-Leader in Safety Tech | UK | Misinformation, Disinformation, Hate Crime, Online Abuse (broadly) |
| [108] | DCMS | NA | 2022 | Influencer Culture: Lights, Cameras, Inaction? | UK | Children and YP as influencers: Online Harassment, Privacy Risk, Child Abuse |
| [79] | Eating Disorders Families Australia | NA | 2022 | Social Media and Online Safety | AUS | Eating Disorders |
| [88] | EKO | NA | 2023 | Suicide, Incels, and Drugs: How TikTok's deadly algorithm harms kids | USA | Suicide & Self-Harm |
| [10] | eSafety Commissioner | NA | 2024 | Tipping the balance - LGBTIQ+ teens' experiences negotiating connection, self-expression and harm online | AUS | Mental Health & Wellbeing |
| [117] | Govt. Communications Headquarters & DCMS | NA | 2020 | VoCO - Verification of Children Online | UK | Childhood Sexual Abuse (CSA) |
| [118] | Health and Social Care Committee | NA | 2022 | The Impact of Body Image on Mental and Physical Health | UK | Body Image, Body Dysmorphia, Eating Disorders, Mental Health and Wellbeing |
| [97] | HOPELAB, Common Sense Media, NORC (University of Chicago) | Mary Madden, Angela Calvin, and Alexa Hasse | 2024 | A Double-Edged Sword: How Diverse Communities of Young People Think about the Multifaceted Relationship Between Social Media and Mental Health | USA | Mental Health and Wellbeing |
| [82] | House of Commons | Women and Equalities Committee | 2021 | Changing the Perfect Picture: An Inquiry into Body Image | UK | Body Image |
| [109] | Inclusion London | NA | 2023 | Tackling Online Abuse | UK | Discrimination, Hate Crime |
| [106] | Information Technology and Innovation Fund | Ashley Johnson | 2024 | How to Address Children’s Online Safety in the United States | USA | CSA |
| [119] | Institute for Strategic Dialogue (ISD) | Chloe Colliver & Jennie King | 2020 | The First 100 Days: Coronavirus and Crisis Management on Social Media Platforms | Global | Misinformation, Disinformation |
| [41] | ISD | Ciarán O’Connor | 2021 | Hatescape: An in-depth analysis of Extremism and Hate Speech on TikTok | Global | Extremism & Hate Speech |
| [39] | ISD | Mauritius Dorn, Sara Bundtzen, Christian Schwieter, & Milan Gandhi | 2023 | Emerging Platforms and Technologies: An Overview of the Current Threat Landscape and its Policy Implications | Global | Misinformation, Disinformation, Extremism, Online harm in general |
| [86] | ISD | Sara Bundtzen | 2023 | Misogynistic Pathways to Radicalisation: Recommended Measures to Assess and Mitigate Online Gender-Based Violence | Global | TFGBV, Misogyny, Extremism, Radicalisation |
| [101] | ISD | Ciarán O’Connor, Jared Holt, Lucy Cooper and Kevin D. Reyes | 2024 | TikTok Series: Policy Recommendations | Global | Extremism & Hate Speech |
| [120] | ISD | Aoife Gallagher, Lucy Cooper, Rhea Bhatnagar, and Cooper Gatewood | 2024 | Pulling Back the Curtain: An Exploration of YouTube's Recommendation Algorithm | Global | Misinformation, Misogynistic Content (spec. Andrew Tate), Sexual Crime |
| [121] | ISD, B’nai B’rith International, & UNESCO | NA | 2022 | Online Antisemitism: A Toolkit for Civil Society | UK | Antisemitism, Extremism, Disinformation |
| [58] | ISD & Reset. Tech Australia | Elise Thomas & Kata Balint | 2022 | Algorithms as a Weapon Against Women: How YouTube Lures Boys and Young Men into the ‘Manosphere | AUS | Misogynistic Content |
| [89] | JED Foundation | NA | 2024 | The Jed Foundation (JED) Recommendations for Safeguarding Youth Well-Being on Social Media Platforms | USA | Mental Health & Wellbeing |
| [100] | Kids Online Health and Safety Task Force | NA | 2024 | Online Health and Safety for Children and Youth: Best Practices for Families and Guidance for Industry | USA | Mental Health & Wellbeing |
| [122] | National Academies of Sciences, Engineering, and Medicine | NA | 2024 | Social Media and Adolescent Health | USA | Mental Health & Wellbeing, Misinformation, & Disinformation |
| [104] | National Centre for Social Research & City, University of London | Sarah Sharrock, Nathan Hudson, Jane Kerr, Charlotte Chalker, Maria David, Carrie-Anne Myers | 2024 | Key attributes and experiences of cyberbullying among children in the UK | UK | Cyberbullying |
| [96] | NYC Health | NA | 2023 | New York City’s Role in the National Crisis of Social Media and Youth Mental Health | USA | Mental Health & Wellbeing |
| [78] | Office of the New York State Attorney General Letitia James | NA | 2022 | Investigative Report on the role of online platforms in the tragic mass shooting in Buffalo | USA | Extremism, Hate Crime, Misinformation |
| [105] | Onward | Luke Stanley, Will Tanner, Jenevieve Treadwell, James Blagden | 2022 | The Kids Aren't Alright: The 4 Factors Driving a Dangerous Detachment from Democracy - Onward | UK | Mental Health & Wellbeing (spec. Loneliness) |
| [98] | Open Technology Institute | Sarah Forland, Nat Meysenburg, & Erika Solis | 2024 | Age Verification: The Complicated Effort to Protect Youth Online | USA | Mental Health & Wellbeing |
| [123] | Orygen | NA | 2024 | Responding to the Social and Economic Drivers of Youth Mental Health | AUS | Mental Health & Wellbeing |
| [110] | Orygen & Headspace | NA | 2024 | Social Media and Australian Society: Orygen & Headspace Submission | AUS | Mental Health & Wellbeing |
| [107] | Parliament of the Commonwealth of Australia | House of Representatives Standing Committee on Social Policy and Legal Affairs | 2020 | Protecting the age of innocence | AUS | Online Pornography |
| [124] | Parliament of the Commonwealth of Australia | Economics References Committee | 2023 | Influence of International Digital Platforms | AUS | Misinformation, Disinformation, CSA |
| [103] | Prevention United | Maidment, K, Tonna, Z, Houlihan, M, and Carbone, S. | 2024 | The Impact of Screen Time and Social Media on the Mental Health of Young Australians | AUS | Mental Health & Wellbeing |
| [77] | Public Policy Forum | NA | 2022 | Canadian Commission on Democratic Expression: How to Make Online Platforms More Transparent and Accountable to Canadian Users | CANADA | Extremism, Disinformation, Misinformation |
| [90] | RAND Europe | Kate Cox, Theodora Ogden, Victoria Jordan, Pauline Paille | 2021 | COVID-19, Disinformation and Hateful Extremism | UK | Extremism |
| [125] | ReachOut | Camilla Chaudhary, | 2024 | Harnessing the Feed | AUS | Mental Health & Wellbeing |
| [52] | ReachOut, Beyond Blue, & Black Dog Institute | NA | 2024 | Inquiry into social media impacts on Australian society | AUS | Mental Health & Wellbeing |
| [126] | Reset. Tech Australia | Dylan Williams, Alexandra McIntosh, & Rys Farthing | 2021 | Profiling Children for Advertising: Facebook’s Monetisation of Young People’s Personal Data | AUS | Mental Health & Wellbeing, Misinformation, Pornography |
| [127] | Reset. Tech Australia | NA | 2022 | The Future of Digital Regulation in Australia: Five Policy Principles for a Safer Digital World | AUS | Mental Health & Wellbeing, Misinformation, & Disinformation |
| [54] | Reset. Tech Australia | Rys Farthing | 2022 | Designing for Disorder: Algorithms amplify pro-anorexia content to teens and children as young at 10 | AUS | Eating Disorder, Body Image, Body Dysmorphia |
| [65] | Reset. Tech Australia | NA | 2024 | A duty of care in Australia's Online Safety Act | AUS | Mental Health & Wellbeing |
| [84] | Reset. Tech Australia | NA | 2024 | Achieving Digital Platform Public Transparency in Australia | AUS | Misinformation & Disinformation |
| [111] | Reset. Tech Australia | NA | 2024 | Best Interests & Targeting: Implementing the Privacy Act Review to advance children's rights | AUS | Mental Health & Wellbeing |
| [128] | Reset. Tech Australia | NA | 2024 | Digital Platform Regulation Green Paper | AUS | Mental Health & Wellbeing, Misinformation, Disinformation, & CSA |
| [69] | Reset. Tech Australia & Hannah Jarman | NA | 2024 | Not Just Algorithms: Assuring User Safety Online with Systemic Regulatory Frameworks | AUS | Eating Disorders |
| [99] | Reset. Tech Australia, ChildFund Australia, and Australian Child Rights Taskforce | Rys Farthing | 2022 | How outdated approaches to regulation harm children and young people and why Australia urgently needs to pivot | AUS | Mental Health & Wellbeing, Misinformation, Disinformation, & CSA |
| [93] | Royal College of Psychiatrists (RCPSYCH) | Bernadka Dubicka & Louise Theodosiou | 2020 | Technology use and the mental health of children and young people | UK | Cyberbullying, Self-harm, Suicide and Body Image |
| [83] | Royal Society for Arts (RSA) | Asheem Singh & Jake Jooshandeh | 2021 | Platforms and the Public Square: A taxonomy of misinformation and the misinformed | Global | Misinformation, Disinformation |
| [129] | Samaritans | NA | 2022 | Towards a Suicide-Safer Internet | UK | Suicidal Ideation and Self-harm |
| [31] | Scottish Government and Mental Health Foundation | NA | 2020 | Body Image: We are more than what we look like | UK | Body Image |
| [130] | The Centre for Social Justice | NA | 2021 | Unsafe Children: Driving up our country’s response to child sexual abuse and exploitation | UK | CSA |
| [102] | The Office of Minnesota Attorney General Keith Ellison | NA | 2024 | Minnesota Attorney General's Report on Emerging Technology and its Effects on Youth Well-Being | USA | Mental Health & Wellbeing, CSA |
| [131] | UNICEF Australia | NA | 2024 | Protecting Children in the Online World | AUS | Mental Health & Wellbeing, Cyberbullying |
| [94] | US Surgeon General | NA | 2021 | Protecting Youth Mental Health | USA | Mental Health & Wellbeing |
| [132] | US Surgeon General | NA | 2023 | Our Epidemic of Loneliness and Isolation | USA | Depression, Anxiety, Suicidality, and Self-harm |
| [3] | US Surgeon General | NA | 2023 | Social Media and Youth Mental Health | USA | Mental Health & Wellbeing |
| [133] | WHO | NA | 2024 | Teens, Screens and Mental Health | Global | Mental Health & Wellbeing |
| [134] | ySKILLS | Sonia Livingstone, Mariya Stoilova, Line Indrevoll Stanicke, Reider Schei Jessen, Richard Graham, Elisabeth Staksurd, Tine Jensen | 2022 | Young People Experiencing Internet-Related Mental Health Difficulties: The Benefits and Risks of Digital Skills | UK | Mental Health and Wellbeing |

References

3. Social media and youth mental health. The U.S. Surgeon General's Advisory. URL: <https://www.hhs.gov/sites/default/files/sg-youth-mental-health-social-media-advisory.pdf> [accessed 2025-01-31].

10. Tipping the balance. LGBTQI+ teens’ experiences negotiating connection, self-expression and harm online. eSafety Commissioner. URL: <https://www.esafety.gov.au/sites/default/files/2024-06/Tipping-the-balance-June-2024.pdf> [accessed 2025-05-29]

31. Body image: we are more than what we look like. Scottish Government's Body Image Advisory Group on Good Body Image & Mental Health Foundation. URL: <https://www.mentalhealth.org.uk/sites/default/files/2022-06/>

33. Driven into darkness: how TikTok’s ‘for you’ feed encourages self-harm and suicidal ideation. Amnesty International. <URL:https://www.amnesty.org/en/documents/POL40/7350/2023/en/> [accessed 2025-01-31]

39. Dorn M, Bundtzen S, Schwieter C, Gandhi M. Emerging platforms and technologies: an overview of the current threat landscape and its policy implications. Institute for Strategic Dialogue. URL: <https://www.isdglobal.org/wp-content/uploads/2023/10/Emerging-Platforms-and-Technologies-An-Overview-of-the-Current-Threat-Landscape-and-its-Policy-Implications.pdf> [accessed 2025-01-31]

41. O'Connor C. Hatescape: an in-depth analysis of extremism and hate speech on TikTok. Institute for Strategic Dialogue. URL: <https://www.isdglobal.org/wpcontent/uploads/2021/08/HateScape_v5.pdf> [accessed 2025-05-29]

52. Inquiry into social media impacts on Australian Society. Joint Select Committee on Social Media and Australian Society Submission 168. URL: <https://www.blackdoginstitute.org.au/wp-content/uploads/2024/07/Sub168_ReachOut-Beyonce-Blue-BDI.pdf> [accessed 2025-01-31]

54. Farthing R. Designing for disorder: algorithms amplify pro-anorexia content to teens and children as young. Reset Australia. URL: <https://au.reset.tech/uploads/insta-pro-eating-disorder-bubble-april-22-1.pdf> [accessed 2025-01-31]

56. Adolescent screen use and mental health: summary of findings from the Future Proofing Study. Black Dog Institute. URL: <https://www.blackdoginstitute.org.au/research-projects/teens-screens-adolescent-mental-health-screen-use/> [accessed 2025-05-29]

65. A duty of care in Australia's online safety act. Reset Australia. URL: <https://au.reset.tech/uploads/Duty-of-Care-Report-Reset.Tech.pdf> [accessed 2025-01-31]

69. Not just algorithms: assuring user safety online with systemic regulatory frameworks. Analysis & Policy Observatory. URL: <https://apo.org.au/sites/default/files/resource-files/2024-03/apo-nid326122.pdf> [accessed 2025-01-31]

77. How to make online platforms more transparent and accountable to Canadian users. Canadian Commission on Democratic Expression. URL: <https://static1.squarespace.com/static/5ea874746663b45e14a384a4/t/62794492003e16063fab7552/1652114580390/DemX+2+-+English+-+May+4.pdf> [accessed 2025-01-31]

78. Investigative report on the role of online platforms in the tragic mass shooting in buffalo on May 14, 2022. Office of the New York State Attorney General Letitia James. URL: <https://ag.ny.gov/sites/default/files/buffaloshooting-onlineplatformsreport.pdf> [accessed 2025-01-31]

79. Inquiry into social media and online safety submission 37. Eating Disorders Families Australia. URL: <https://www.aph.gov.au/Parliamentary_Business/Committees/House/Former_Committees/Social_Media_and_Online_Safety/SocialMediaandSafety/Submissions> [accessed 2025-01-31]

80. Patnaik S, Litan RE. TikTok shows why social media companies need more regulation. Center on Regulation & Markets.URL: <https://www.brookings.edu/wp-content/uploads/2023/05/20230511_CRM_PatnaikLitan_TikTok_FINAL.pdf> [accessed

2025-01-31]

81. Social media, body image, and eating disorders. Butterfly Foundation. URL: <https://butterfly.org.au/wp-content/uploads/2024/05/Roundtable-Recommendations-FINAL-ONLINE.pdf> [accessed 2025-01-31]

82. Changing the perfect picture: an inquiry into body image. Women and Equalities Committee. URL: <https://dera.ioe.ac.uk/id/eprint/37748/1/download%20%281%29.pdf> [accessed 2025-01-31]

83. Singh A, Jooshandeh J. Platforms and the public square: a taxonomy of misinformation and the misinformed. Royal Society for Arts & BT Group. URL: <https://www.thersa.org/globalassets/_foundation/new-site-blocks-and-images/reports/2021/11/platforms_and_the_public_square.pdf> [accessed 2025-01-31]

84. Achieving digital platform public transparency in Australia. Reset Australia. URL: <https://au.reset.tech/Digital-platform-public-transparency.pdf> [accessed 2025-01-31]

85. 'I feel exposed': caught in TikTok's surveillance web. Amnesty International. URL: <https://www.amnesty.org/en/documents/pol40/7349/2023/en/> [accessed 2025-01-31]

87. Online targeting: final report and recommendations. Centre for Data Ethics and Innovation. URL: <https://www.gov.uk/government/publications/cdei-review-of-online-targeting/online-targeting-final-report-and-recommendations#introduction> [accessed 2025-01-31]

90. Cox K, Ogden T, Jordan V, Paille P. COVID-19, disinformation and hateful extremism. RAND Europe. URL: <https://assets.publishing.service.gov.uk/media/60c868448fa8f57ce58ce901/RAND_Europe_Final_Report_Hateful_Extremism_During_COVID-19_Final.pdf> [accessed 2025-01-31]

91. Bradshaw K, Vaillancourt T. Freedom of thought, social media and the teen brain. Centre for International Governance Innovation. URL: <https://www.cigionline.org/static/documents/FoT_PB_no.9.pdf> [accessed 2025-01-31]

92. Health advisory on social media use in adolescence. American Psychological Association. URL: <https://www.apa.org/topics/social-media-internet/health-advisory-adolescent-social-media-use.pdf> [accessed 2025-01-31]

93. Dubicka B, Theodosiou L. Technology Use and the Mental Health of Children and Young People. Royal College of Psychiatrists. URL: <https://www.rcpsych.ac.uk/docs/default-source/improving-care/better-mh-policy/college-reports/college-report-cr225.pdf> [accessed 2025-01-31]

94. Protecting youth mental health. The U.S. Surgeon General's Advisory. URL: <https://www.hhs.gov/sites/default/files/surgeon-general-youth-mental-health-advisory.pdf> [accessed 2025-01-31]

95. Diepeveen S. How does social media influence gender norms among adolescent boys? Key evidence and policy implications. Advancing Learning and Innovation on Gender Norms (ALiGN). URL: <https://www.alignplatform.org/sites/default/files/2024-02/align-socialmedia-briefingnote-jan24-proof05.pdf> [accessed 2025-01-31]

96. New York City’s role in the national crisis of social media and youth mental health: framework for action. NYC Health. URL: <https://www.nyc.gov/assets/doh/downloads/pdf/mh/social-media-youth-mental-health-framework-action.pdf> [accessed 2025-01-31]

97. Madden M, Calvin A, Hasse A. A double-edged sword: how diverse communities of young people think about the multifaceted relationship between social media and mental health. Common Sense Media & HopeLab. URL: <https://www.commonsensemedia.org/sites/default/files/research/report/2024-double-edged-sword-hopelab-report_final-release-for-web-v2.pdf> [accessed 2025-01-31]

98. Forland S, Meysenburg N, Solis E. Age verification: the complicated effort to protect youth online. Open Technology Institute. URL: <https://www.newamerica.org/oti/reports/age-verification-the-complicated-effort-to-protect-youth-online/> [accessed 2025-01-31]

99. Farthing R. How outdated approaches to regulation harm children and young people and why Australia urgently needs to pivot. Reset Australia, ChildFund Australia, & Australian Child Rights Taskforce. URL: <https://au.reset.tech/uploads/insta-pro-eating-disorder-bubble-april-22-1.pdf> [accessed 2025-01-31]

100. Online health and safety for children and youth: best practices for families and guidance for industry. Kids Online Health and Safety Task Force. URL: <https://www.samhsa.gov/sites/default/files/online-health-safety-children-youth-report.pdf> [accessed 2025-01-31]

101. TikTok series: policy recommendations. Institute for Strategic Dialogue. URL: <https://www.isdglobal.org/digital_dispatches/trouble-with-tiktok-policy-recommendations/> [accessed 2025-01-31]

102. Minnesota attorney general's report on emerging technology and its effects on youth well-being. The Office of Minnesota Attorney General Keith Ellison. URL: <https://www.lrl.mn.gov/docs/2024/mandated/240175.pdf> [accessed 2025-01-31]

103. Maidment K, Tonna Z, Houlihan M, Carbone S. The impact of screen time and social media on the mental health of young Australians. Melbourne: Prevention United. URL: <https://nest.greenant.net/index.php/s/QiR56KZpQzMPPBn?mc_cid=8b145254ee&mc_eid=67df8ff35d> [accessed 2025-01-31]

104. Sharrock S, Hudson N, Kerr J, Chalker C, David M, Myers C. Key attributes experiences of cyberbullying among children in the UK. National Centre for Social Research. URL: <https://www.ofcom.org.uk/siteassets/resources/documents/research-and-data/online-research/keeping-children-safe-online/experiences-of-children/key-attributes-and-experiences-of-cyberbullying-among-children-in-the-uk.pdf?v=368017#:~:text=Repetition%20and%20intentionality%20were%20described,some%20participants%20as%20sometimes%20challenging> [accessed 2025-01-31]

105. Stanley L, Tanner W, Treadwell J, Blagden J. The kids aren't alright: the 4 factors driving a dangerous detachment from democracy. UK Onward. URL: <https://www.ukonward.com/reports/the-kids-arent-alright-democracy/> [accessed 2025-01-31]

106. Johnson A. How to address children's online safety in the United States. Information Technology & Innovation Foundation. URL: <https://www2.itif.org/2024-child-online-safety.pdf> [accessed 2025-01-31]

107. Protecting the age of innocence. House of Representatives Standing Committee on Social Policy and Legal Affairs. URL: <https://www.aph.gov.au/Parliamentary_Business/Committees/House/Social_Policy_and_Legal_Affairs/Onlineageverification/Report> [accessed 2025-01-31]

108. Influencer culture: lights, camera, inaction. Digital, Culture, Media and Sport Committee. URL: <https://publications.parliament.uk/pa/cm5802/cmselect/cmcumeds/258/report.html> [accessed 2025-01-31]

109. Tackling online abuse: petitions commons select committee’s inquiry on online abuse and the experience of disabled people. Inclusion London. URL: <https://committees.parliament.uk/writtenevidence/9813/pdf/> [accessed 2025-01-31]

110. Social media and Australian society. Orygen Institute & Headspace. URL: <https://headspace.org.au/assets/Advocacy-and-policy-submissions/Orygen-headspace-submission-Joint-Select-Committee-on-Social-Media-and-Australian-Society_v2.pdf> [accessed

2025-01-31]

111. Best interests and targeting: implementing the privacy act review to advance children's rights. Reset Australia. URL: <https://au.reset.tech/uploads/Best-Interests-Report-240128-digital.pdf> [accessed 2025-01-31]

112. Galea S, Buckley G. Social media and adolescent mental health: a consensus report of the National Academies of Sciences, Engineering, and Medicine. PNAS Nexus. Mar 2024;3(2):pgae037. [FREE Full text] [doi: 10.1093/pnasnexus/pgae037] [Medline: 38415222]

113. The incelosphere: exposing pathways into incel communities and the harms they pose to women and children. Center for Countering Digital Hate Quant Lab. URL: <https://counterhate.com/wp-content/uploads/2023/08/CCDH-The-Incelosphere-FINAL.pdf> [accessed 2025-01-31]

114. Dunn S, Vaillancourt T, Brittain H. Supporting safer digital spaces. Centre for International Governance Innovation. URL: <https://www.cigionline.org/static/documents/SaferInternet_Special_Report.pdf> [accessed 2025-01-31]

115. Lenhart A, Owens K. The unseen teen - the challenges of building healthy tech for young people. Data and Society. URL: <https://datasociety.net/library/the-unseen-teen/> [accessed 2025-01-31]

116. Perspective Economics, Aiken M, Davidson J. Safer technology, safer users: the UK as a world-leader in safety tech. University of East London. URL: <https://assets.publishing.service.gov.uk/media/60622469e90e072d9af1df16/Safer_technology__safer_users-_The_UK_as_a_world-leader_in_Safety_Tech_V2.pdf> [accessed 2025-01-31]

117. VoCO - verification of children online phase 2 report. Government Communications Headquarters; Department for Digital, Culture, Media & Sport. URL: <https://assets.publishing.service.gov.uk/media/5faa9cffd3bf7f03a841cfc2/November_VoCO_report_V4__pdf.pdf> [accessed 2025-01-10]

118. The impact of body image on mental and physical health. Health and Social Care Committee. URL: <https://committees.parliament.uk/publications/23284/documents/170077/default/> [accessed 2025-01-10]

119. Colliver C, King J. The first 100 days: coronavirus crisis management on social media platforms. Institute for Strategic Dialogue. URL: <https://www.isdglobal.org/wp-content/uploads/2020/06/First-100-Days.pdf> [accessed 2025-01-10]

120. Gallagher A, Cooper L, Bhatnagar R, Gatewood C. Pulling back the curtain: an exploration of YouTube's recommendation algorithm. Institute for Strategic Dialogue. URL: <https://www.isdglobal.org/wp-content/uploads/2024/06/Pulling-Back-the-Curtain-Executive-Summary-6.pdf> [accessed 2025-01-31]

121. Online antisemitism: a toolkit for civil society. Institute for Strategic Dialogue. URL: <https://unesdoc.unesco.org/ark:/48223/pf0000381856> [accessed 2025-01-10]

122. Social media and adolescent health. National Academies of Sciences, Engineering, and Medicine. URL: <https://doi.org/10.17226/27396> [accessed 2025-01-31]

123. Responding to the social and economic drivers of youth mental health: policy lab. Orygen Institute. URL: <https://www.orygen.org.au/Orygen-Institute/Policy-Areas/Social-and-environmental-factors/2024-Policy-Lab-responding-social-economic-drivers.aspx?ext=.pdf> [accessed 2025-01-10]

124. Influence of international digital platforms. Economics References Committee. URL: <https://parlinfo.aph.gov.au/parlInfo/download/committees/reportsen/RB000119/toc_pdf/Influenceofinternationaldigitalplatforms.pdf> [accessed 2025-01-31]

125. Chaudhary C. Harnessing the feed: social media for mental health information and support. ReachOut. URL: <https://d1robvhmkdqpun.cloudfront.net/46e49639306f108e035212644ba15d45.pdf> [accessed 2025-01-31]

126. Williams D, McIntosh A, Farthing R. Profiling children for advertising: Facebook’s monetisation of young people’s personal data. Reset Australia. URL: <https://au.reset.tech/uploads/resettechaustralia_profiling-children-for-advertising-1.pdf> [accessed

2025-01-31]

127. The future of digital regulation in Australia: five policy principles for a safer digital world. Reset Australia. URL: <https://au.reset.tech/uploads/the-future-of-digital-regulations-in-australia.pdf> [accessed 2025-01-31]

128. Digital platform regulation green paper. Reset Australia. URL: <https://au.reset.tech/uploads/Digital-Platform-Regulation-Green-Paper.pdf> [accessed 2025-01-31]

129. Towards a suicide-safer internet. Samaritans. URL: <https://nspa.org.uk/wp-content/uploads/2022/02/Samaritans_WhatASafeInternetLooksLike_2022.pdf> [accessed 2025-01-31]

130. Unsafe children: driving up our country's response to child sexual abuse and exploitation. The Centre for Social Justice. URL: <https://www.centreforsocialjustice.org.uk/wp-content/uploads/2021/03/CSJJ8804-Unsafe-Children-210325-WEB.pdf> [accessed 2025-01-31]

131. Protecting children in the online world. UNICEF Australia. URL: <https://assets-us-01.kc-usercontent.com/99f113b4-e5f7-00d2-23c0-c83ca2e4cfa2/6cde226b-23d1-413a-bac3-7f0eafe524d4/UA_Digital-Wellbeing-Position-Paper-2024_LR_FINAL.pdf> [accessed 2025-01-31]

132. Our epidemic of loneliness and isolation. The U.S. Surgeon General's Advisory, US Department of Health and Human Services. URL: <https://www.hhs.gov/sites/default/files/sg-youth-mental-health-social-media-advisory.pdf> [accessed 2025-01-31]

133. Teens, screens and mental health. World Health Organization. URL: <https://www.who.int/europe/news-room/25-09-2024-teens--screens-and-mental-health> [accessed 2025-01-31]

134. Livingstone S, Stoilova M, Stänicke L. Young people experiencing internet-related mental health difficulties: the benefits and risks of digital skills. An empirical study. ySkills. URL: <https://eprints.lse.ac.uk/116407/3/D6.1_ySkills_WP6.4_Mental_health_difficulties_and_digital_skills_Report_Final.pdf> [accessed 2025-05-29]
